# Circular RNA circ_0090231 promotes atherosclerosis in vitro by enhancing NLR family pyrin domain containing 3-mediated pyroptosis of endothelial cells

**DOI:** 10.1080/21655979.2021.1989260

**Published:** 2021-11-30

**Authors:** Yishan Ge, Wenwu Liu, Wei Yin, Xuebin Wang, Jie Wang, Xiaoqing Zhu, Shengkai Xu

**Affiliations:** Department of Cardiology, Affiliated Suzhou Science and Technology City Hospital of Nanjing Medical University, Suzhou, China

**Keywords:** Atherosclerosis, circ_0090231, pyroptosis, NLRP3, miR-635

## Abstract

Atherosclerosis (AS) is an inflammatory disease caused by multiple factors. Multiple circRNAs are involved in the development of AS. The present study focusses on delineating the role of circ_0090231 in AS. Human aortic endothelial cells (HAECs) were treated with oxidized low-density lipoprotein (ox-LDL) to construct an *in vitro* AS model. Real-time quantitative polymerase-chain reaction (RT-qPCR) was used to detect the levels of circ_0090231, IL-1β, and IL-18 transcripts. CircRNA/target gene interactions were predicted using StarBase and TargetScan and confirmed using an RNA pull-down assay and dual-luciferase reporter assay. Further, 3-(4,5)-dimethylthiahiazo(−2)-3,5-diphenytetrazoliumromide (MTT) and lactate dehydrogenase (LDH) release assays were performed to evaluate cell viability and damage in the AS model, respectively. Cell pyroptosis and protein expression were determined using flow cytometry and western blotting respectively. The treatment of HAECs with ox-LDL not only led to significant increase in the levels of circ_0090231 but also resulted in improved cell viability as well as reduced cell injury and pyroptosis as compared to that in non-treated cells. The circ_0090231 was also identified to function as a sponge for miR-635, knockdown of which reverses the effects of circ_0090231 inhibition. Furthermore, our results revealed that levels of NLRP3, a miR-635 target, are not only augmented in the AS model but its overexpression also weakens the miR-635 regulatory effects in the AS development. Taken together, the circ_0090231/miR-635/NLRP3 axis affects the development of AS by regulating cell pyroptosis, thus providing new insights into the mechanism of AS development.

## 1. Introduction

Cardiovascular diseases are the leading cause of morbidity and mortality worldwide [1]. Atherosclerosis (AS) is the main pathological basis of coronary heart disease, stroke, and other cardiovascular diseases [2]. The development of AS is a complex pathological process that is attributed to various phenomena such as immune and injury inflammation, platelet activation, and oxidative stress theory [3–5]. With recent advances in the field of AS research, various chemical factors and cytokines have been identified in the pathogenesis of this condition. In recent years, an increasing number of mainstream interpretations indicate that AS is an inflammatory disease with a general pattern of inflammatory manifestations [6,7]. As a result, elucidating the molecular mechanisms regulating the inflammatory response during AS is of vital importance.

Pyroptosis was first proposed by Zychlinsky et al. in 1992 [8]. It is a novel form of pro-inflammatory programmed cell death, which is mediated by Caspase 1 and accompanied by the release of numerous pro-inflammatory factors (such as interleukin (IL) 1β and IL-18) to induce an amplified inflammatory cascade [9]. Gasdermin D (GSDMD) is a substrate of Caspase 1, and its cleavage causes pyroptosis by forming pores in the cell membrane [10,11]. It has been reported that the pathogenesis of AS is associated with the pyroptosis of vascular endothelial cells, macrophages, as well as smooth muscle cells [12,13]. Inhibiting pyroptosis through drug or genetic intervention has been shown to exert a protective effect on AS [14,15]. Thus, the phenomenon of pyroptosis plays a vital role in the pathogenesis of AS.

Circular RNAs (circRNAs) are covalently bonded closed circular RNA molecules without a 5′-terminal cap and 3′-terminal poly (A) tail structure [16]. CircRNAs have been documented to function as competitive endogenous RNAs (ceRNAs), as they are rich in miRNA response elements (MREs) [17]. These MREs enables circRNAs to function as a ‘sponge’ for miRNAs by adsorbing specific target miRNAs, thereby regulating their functions [18]. It has been reported that a variety of circRNAs are present in patients with AS [19]. *In vivo* animal studies have shown that circRNAs act as miR-specific ceRNAs in the pathogenesis of AS by regulating their target mRNA expressions [20]. While there are convincing evidences that circRNAs play an important role in the occurrence and development of AS, there are only few studies that have attempted to delineate their role comprehensively.

In the present study, we aimed to explore the clinical relevance of circ_0090231 in AS, as well as the targeted association between miR-635 and NLRP3 pathway. We hypothesized circ_0090231 regulated pyroptosis and promoted AS via the miR-365/NLRP3 axis. Our findings may provide new evidence in terms of AS prevention and therapy.

## 2. Materials and methods

### 2.1. Cell culture and transfection

HAECs were purchased from the Type Culture Collection of the Chinese Academy of Sciences (Shanghai, P. R. China). Endothelial cell medium with endothelial cell growth factors, 5% fetal bovine serum, 100 U/mL penicillin, and 100 mg/mL streptomycin (Gibco, MA, USA) was used to culture the cells at 37°C with 5% CO_2_.

HAECs were treated with 25 µg/mL ox-LDL (Beijing Xiesheng Bio-Technology Ltd., Beijing, P. R. China) for 24 h to establish an *in vitro* AS model.

Small interference RNA specific to circ_0090231 (si-circ_0090231), miR-635 mimics/inhibitor, NLRP3, and negative controls (Abiocenter Biotech Co., Ltd., Beijing, P. R. China) were transfected into the cells using Lipofectamine® 2000 reagent (Invitrogen; Thermo Fisher Scientific Inc., MA, USA) according to the manufacturer’s protocols at 37°C with 5% CO_2_. After 48 h of transfection, cells were used in subsequent experiments.

### 2.2. Cell viability assay

The cell viability was detected according to a previous study [21]. HAECs (1 × 10^4^ cells/mL) were seeded into 96-well cell culture plates. After indicated treatments, 20 μL MTT reagent (AMJ-KT0001; AmyJet Technology Co., Ltd., Hubei, China) was added to each well of the plate and the cells were cultured at 37°C with 5% CO_2_ for 4 h. Subsequently, a microplate reader (HBS-1096 C; Nanjing DeTie Experimental Equipment Co., Ltd., Jiangsu, China) was used to measure the absorbance of tetratzolium salts from MTT assay at 490 nm.

### 2.3. Lactate dehydrogenase (LDH) release assay

LDH was detected according to a previous study [22]. HAECs (1 × 10^4^ cells/mL) were cultured in 96-well plates at 37°C with 5% CO_2_ and treated with ox-LDL (100 μg/mL) for 48 h, and the LDH content was measured using an LDH activity assay kit (Beyotime, Jiangsu, P. R. China).

### 2.4. Real-time quantitative polymerase chain reaction (RT-qPCR)

Total RNA was extracted using the TRIzol® reagent (Invitrogen; Thermo Fisher Scientific, Inc.). Reverse transcription and qPCR were performed using a BlazeTaq One-Step SYBR Green RT-qPCR Kit (with ROX) (QP071; GeneCopoeia, Inc., MD, USA) on a SEDI Thermo Cycler controlled by the Control Bus Net software package (Wealtec Bioscience Co., Ltd., New Taipei City, China). All primers were designed and synthesized by Nanjing Genscript Biotech Co., Ltd., (Jianngsu, China) and GAPDH was used as an internal reference. The results were analyzed using the 2^−ΔΔCt^ method. The sequences of the primers were as follows:

circ_0090231: F: 5ʹ-GGAGCTATGTGTGGCCAAGT-3ʹ, R: 5ʹ-CGAGGATCTGGAGAACGAGC-3ʹ; IL-1β, F: 5′-GCAGGCAGTATCACTCATTGTGG-3′, R: 5′-GAGTCACAGAGGATGGGCTCTTC-3′; IL-18, F: 5′-ACCCCAGAAGAGAGGGAGTC-3′, R: 5′-GTAGATGGTGGAATCGGCGT-3′; miR-635, F: 5′-ACTTGGGCACTGAAACAATGTCC-3′, R: 5′- GCTGTCAACGATACGCTACGTAACG-3′; NLRP3, F: 5′-AAACGACCTTCATCCCCACC-3′, R: 5′-CAGGACTGCCCTCCTCTAGT-3′; GAPDH, F: 5′-TCTTGTGCAGTGCCAGCCT-3′, R: 5′-TGAGGTCAATGAAGGGGTCG-3′.

### 2.5. Flow cytometry assay

The pyroptosis of HAECs was determined using the TransDetect® Annexin V-FITC/PI Kit (FA101-01; TransGen Biotech Co., Ltd., Beijing, China). Annexin V-FITC (5 μL) was added to HAECs seeded in 6-well cell culture plates and incubated at room temperature for 15 min in the dark. NovoCyte Advanteon B4 Flow Cytometer and NovoSampler Q software (Agilent Technologies Co., Ltd., CA, USA) were used for flow cytometry data acquisition and analyses.

### 2.6. Western blot analysis

Western blot was described as described previously [23]. After rinsing the cells treated under indicated conditions with pre-chilled PBS solution, the cell pellets were resuspended in RIPA lysis buffer for 30 min for the extraction of total protein. Protein concentration was measured using a BCA protein Assay Kit (Beyotime Biotechnology, Jiangsu, China). Next, electrophoresis was performed to resolve the proteins with 10% SDS-PAGE at 120 V until the bromophenol blue dye front reached the separation adhesive base. The proteins were then transferred on to PVDF membranes (Millipore Sigma., MA, USA) in an icebox at 100 V for 1.5 h. The membranes were then blocked in blocking buffer for 1 h at 4°C. After washing with TBST solution, the membranes were incubated with primary antibodies including anti-pro-Caspase 1, anti-cleaved (CL)- Caspase 1, anti-GSDMD (1:1000; Abcam., MA, USA), and anti-GAPDH (1:3000, Leading Biology Inc., CA, USA) on a rocker at 4°C overnight followed by incubation with appropriate secondary antibodies (1:2000, MultiSciences, Shanghai, China) at room temperature for 2 h. Finally, the protein bands were visualized using an ECL detection system (Thermo Fisher Scientific, Inc., MA, USA).

### 2.7. Dual luciferase reporter assay

The Luciferase reporter assay was carried out according to Unal’s study [24]. The luciferase reporter vectors for the wild-type (WT) and mutant (MUT) 3′-UTR regions of circ_0090231 and NLRP3 were designed and synthesized by Guangzhou RiboBio Co., Ltd., Guangzhou, China. The HAECs were transfected with these vectors culturing for 24 h. The luciferase activities were detected using the Luciferase Reporter Assay Kit (K801-200; BioVision Tech Co., Ltd., Seoul, South Korea) 48 h after co-transfection with miR-635 mimic/control as well as the luciferase reporter vectors. The results were analyzed using a luciferase assay kit (Promega). The luciferase activity was normalized to Renilla luciferase activity.

### 2.8. RNA pull-down assay

RNA pull-down assay was described as described previously [25]. RNA pull-down assay was carried out in accordance to the manufacturer’s protocol using the MagCapture RNA Pull Down Assay Kit (297–77,501; Whatman Co., Ltd., Metestone, UK). The pulled-down proteins were further subjected to mass spectrometry analysis.

### 2.9. Statistical analysis

Each experiment was performed at least three times. GraphPad Prism (version 7, GraphPad Software Inc.) was used to calculate all data, which are presented as the mean ± SD. The Student’s unpaired t-test was applied to compare the differences between two groups, and the differences among multiple groups were analyzed using analysis of variance (ANOVA) followed by Duncan’s post-hoc test. *P* values of <0.05 were considered to suggest a significant difference.

## 3. Results

This study aimed to explore the role of circ_0090231 in AS. We demonstrated that circ_0090231 was up-regulated in the AS cells, and promoting the cell injury and pyroptosis via miR-635/NLRP3 axis.

### 3.1. Ox-LDL treatment induces pyroptosis in HAECs

**Figure 1.**
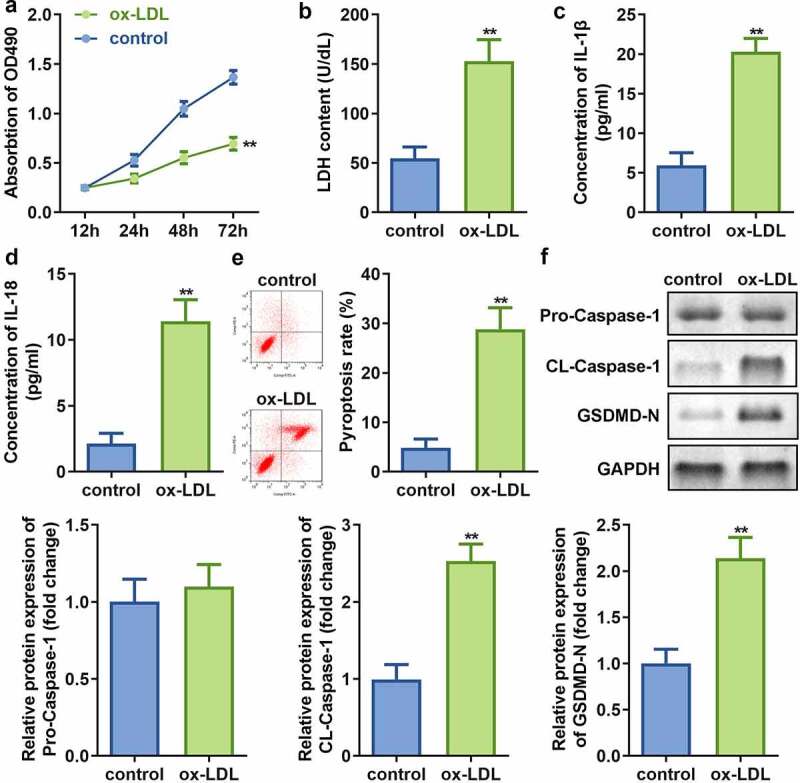
Ox-LDL decreases cell viability and promotes injury along with pyroptosis of HAECs. (a) Cell viability; (b) LDH release; (c) IL-1β levels; (d) IL-18 levels; (e) Cell pyroptosis; and (f) Expression of pro-Caspase 1, cleaved-Caspase 1, and GSDMD in HAECs treated with or without ox-LDL. **P < 0.01 versus control. ox-LDL, oxidized low-density lipoprotein

In order to study the effect of mimicking AS *in vitro*, the HAECs were first exposed to ox-LDL. Subsequent analyses revealed that while ox-LDL treatment of HAECs decreases the cell viability (Figure 1(a)), it also results in significant augmentation of LDH release as compared to that in non-treated control cells (Figure 1(b)). Additionally, the levels of IL-1β and IL-18 in HAECs were found to be significantly increased (Figure 1(c,d)), and induced pyroptosis (Figure 1(e)), which evidenced by cell swelling, large bubbles after treated by ox-LDL (Supplementary, Fig. S1). Moreover, the protein expression of cleaved-Caspase 1 and GSDMD-N was found to be augmented, while no significant difference was observed in pro-Caspase 1 expression (Figure 1(f)) upon ox-LDL treatment of HAECs as compared to the control cells.

### 3.2. HAECs exhibit increased expression of nine pyroptosis associated circRNAs upon treatment with ox-LDL

**Figure 2.**
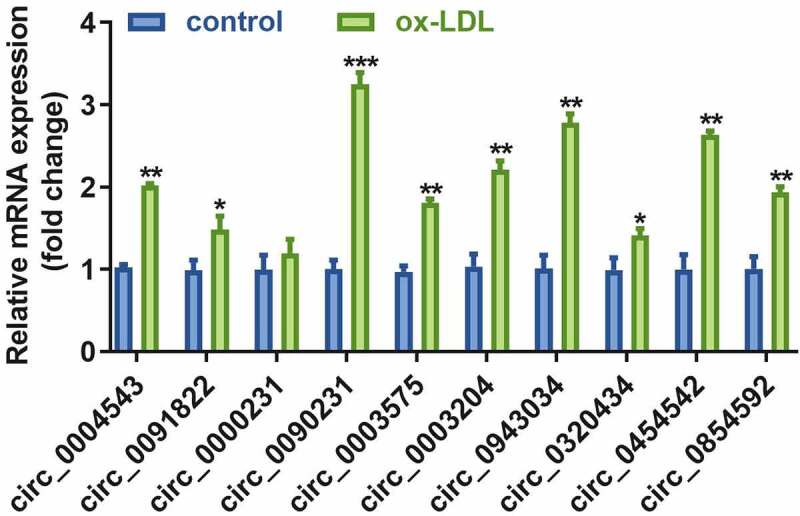
Circ_0090231 is overexpressed in ox-LDL-treated HAECs cells. Expression of circ_0090231 transcripts. *P < 0.05 versus control, **P < 0.01 versus control, ***P < 0.001 versus control

The real-time quantitative PCR results revealed that expression levels of the nine circRNAs were significantly increased in ox-LDL-treated HAECs as compared to control cells (Figure 2). Moreover, the expression of circ_0090231 was higher than other circRNAs.

### 3.3. Inhibition of circ_0090231 expression increases the cell viability and reduces the LDH release and pyroptosis in ox-LDL treated HAECs

**Figure 3.**
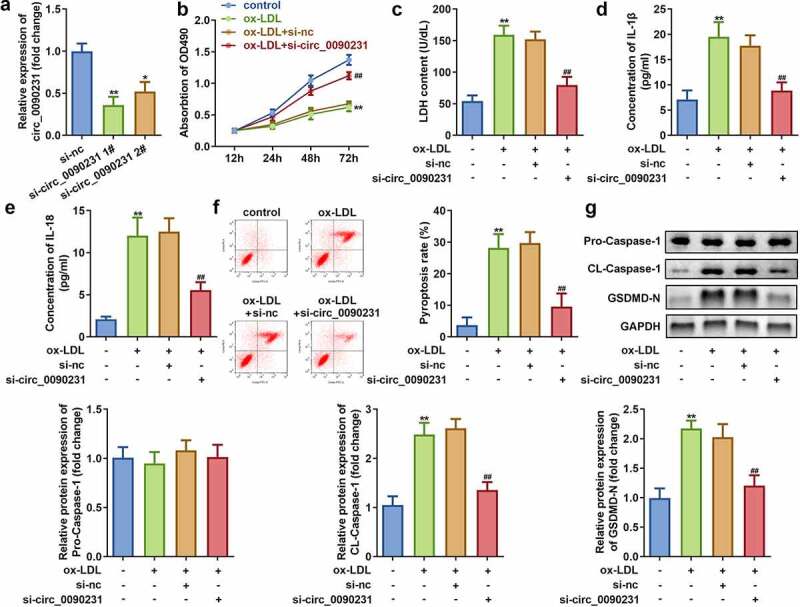
Circ_0090231 knockdown enhances cell viability and suppresses cell injury and pyroptosis in ox-LDL-treated HAECs cells. (a) Expression of circ_0090231; (b) Cell viability; (c) LDH release; (d) IL-1β level; (e) IL-18 level; (f) Cell pyroptosis; and (g) Expression of pro-Caspase 1; cleaved-Caspase 1; GSDMD-N in ox-LDL treated or non-treated HAECs upon transfection with circ_0090231 specific or mock siRNAs. *P < 0.05 versus control, **P < 0.01 versus control, ## *P* < 0.01 versus control. si-nc, blank vector; si- circ_0090231, knockdown of circ_0090231

To examine the effect of circ_0090231 inhibition, the HAECs were first successfully transfected with two circ_0090231 specific siRNAs as indicated by significant decrease in its expression as compared to that in the cells transfected with negative control siRNAs. As the transfection efficiency of circ_0090231 siRNA 1# was more potent, it was used in subsequent experiments (Figure 3(a)). Our results further revealed that inhibition of circ_0090231 significantly improves the cell viability and hinders the LDH release from ox-LDL treated HAECs as compared to that from non-treated control cells (Figure 3(b,c)). In addition, the inhibition of circ_0090231 also results in decreased expression of IL-18 and IL-1β as well as suppression of pyroptosis in HAECs treated with ox-LDL as compared to that in control cells (Figure 3(d–f)). Moreover, the protein expression of cleaved-Caspase 1 and GSDMD-N was also found to be decreased upon circ_0090231 knockdown, while that of pro-Caspase 1 expression remained unaffected in HAECs treated with ox-LDL as compared to that in control cells (Figure 3(g)).

### 3.4. Circ_0090231 directly targets miR-635

**Figure 4.**
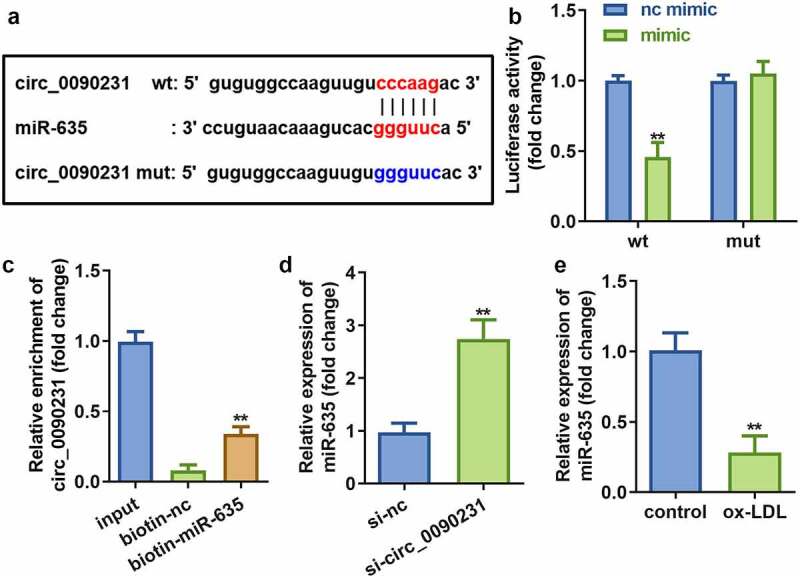
Circ_0090231 sponges miR-635. (a) The binding sites between miR-635 and circ_0090231. (b) The luciferase activity of HAECs cells co-transfected with luciferase reporter vector containing circ_0090231 MREs for miR-635 and miR-635 overexpression vector. (c) The interaction between miR-635 and circ_0090231. (d) Expression of miR-635 in HAECs with circ_0090231 knockdown. (e) Expression of miR-635 in HAECs exposed to ox-LDL vs control cells. **P < 0.01 versus vector, si-NC, biotin-NC or control. si-nc, blank vector; si- circ_0090231, knockdown of circ_0090231

Circ_0090231 has been reported to function as a ceRNA that can regulate various biological processes by sponging miRNAs [26]. We employed Starbase 3.0 (http://starbase.sysu.edu.cn/) to predict potential target miRNAs of circ_0090231. The results revealed that circ_0090231 possesses various MREs for miR-635 (Figure 4(a)). This finding was further validated with the help of dual-luciferase reporter and RNA pull-down assays (Figure 4(b,c)). Furthermore, miR-635 expression was significantly elevated upon circ_0090231 knockdown in ox-LDL treated HAECs as compared to that in control cells (Figure 4(d)). In addition, miR-635 expression was found to be significantly decreased in HAECs treated with ox-LDL as compared to that in control cells (Figure 4(e)).

### 3.5. Inhibition of miR-635 rescues the effects of circ_0090231 on cell viability, injury as well as pyroptosis of HAECs exposed to ox-LDL

**Figure 5.**
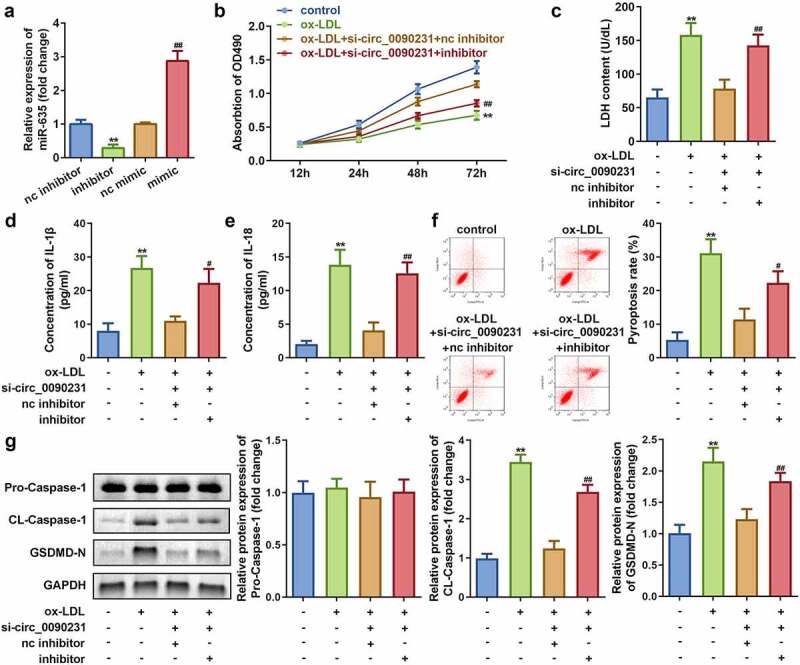
Knockdown of miR-635 inhibits cell viability as well as promotes cell injury and pyroptosis in ox-LDL treated HAECs. (a) Expression of miR-635, (b) Cell viability, (c) LDH release, (d) IL-1β level, (e) IL-18 level, (f) Cell pyroptosis, and (g) Expression of pro-Caspase 1, cleaved-Caspase 1, GSDMD-N in HAECs under indicated conditions. **P < 0.01 versus control, #*P* < 0.05 versus control, ##*P* < 0.01 versus control. inhibitor, knockdown of miR-635; mimic, overexpression of miR-635; si- circ_0090231, knockdown of circ_0090231

To assess the role of miR-635 in the circ_0090231-associated effects on HAECs, miR-635 expression was altered by transfecting either miR-635 mimics or inhibitors in HAECs. Subsequent analyses of these cells revealed that transfection with either miR-635 mimics or inhibitors leads to increased or decreased expression of miR-635, respectively, as compared to that in the mock transfected cells (Figure 5(a)). The results also demonstrated that downregulation of miR-635 attenuates the influence of circ_0090231 knockdown on cell viability and LDH release of ox-LDL treated HAECs as compared to that of the non-treated control cells (Figure 5(b,c)). Meanwhile, the inhibition of miR-635 also rescued the decrease in IL-1β and IL-18 levels and reduction in cell pyroptosis caused by circ_0090231 knockdown (Figure 5(d–f)). In addition, the ox-LDL treated HAECs exhibited increased expression of cleaved-Caspase 1 and GSDMD-N upon inhibition of miR-635 as compared to that in the mock transfected cells (Figure 5(g)).

### 3.6. miR-635 directly targets NLRP3

**Figure 6.**
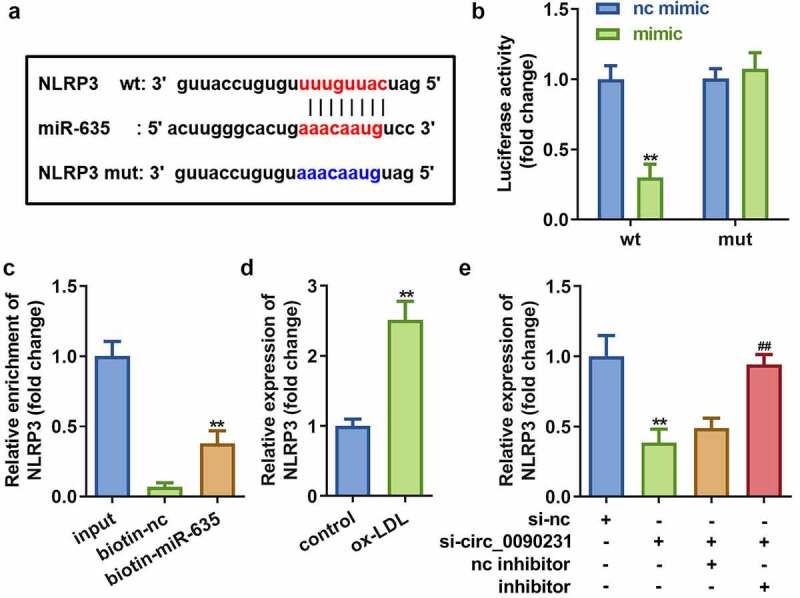
miR-635 directly targets NLRP3. (a) The binding sites of miR-635 on NLRP3 3′-UTR. (b) The luciferase activity of HAECs co-transfected with luciferase reporter vector containing NLRP3 3′-UTR and miR-635 overexpression vector. (c) The interaction between miR-635 and NLRP3. (d) Expression of NLRP3 in HAECs with miR-635 knockdown. (e) Expression of NLRP3 in HAECs cells under indicated conditions. **P < 0.01 versus vector, si-NC, biotin-NC or control. nc inhibitor, blank vector; inhibitor, knockdown of miR-635

To identify specific regulatory pathways involving circ_0090231 and miR-635, TargetScan (http://www.targetscan.org/mamm_31/) was used to predict the target gene of miR-635. Our *in silico* analyses identified NLRP3 as a potential target gene for miR-635 (Figure 6(a)). The dual-luciferase reporter and RNA pull-down assays further confirmed the interaction between NLRP3 and miR-635 as predicted by our *in silico* analysis (Figure 6(b,c)). Furthermore, NLRP3 expressed more in cells exposed to ox-LDL (Figure 6(d)). Meanwhile, the expression of NLRP3 was significantly decreased by circ_0090231 knockdown, but restored to normal level after treatment with miR-635 inhibitor (Figure 6(e)), suggesting that circ_0090231 regulated NLRP3 expression via sponging miR-635.

### 3.7. Overexpression of NLRP3 reverses the effects of miR-635 on cell viability, injury, and pyroptosis in HAECs exposed to ox-LDL

**Figure 7.**
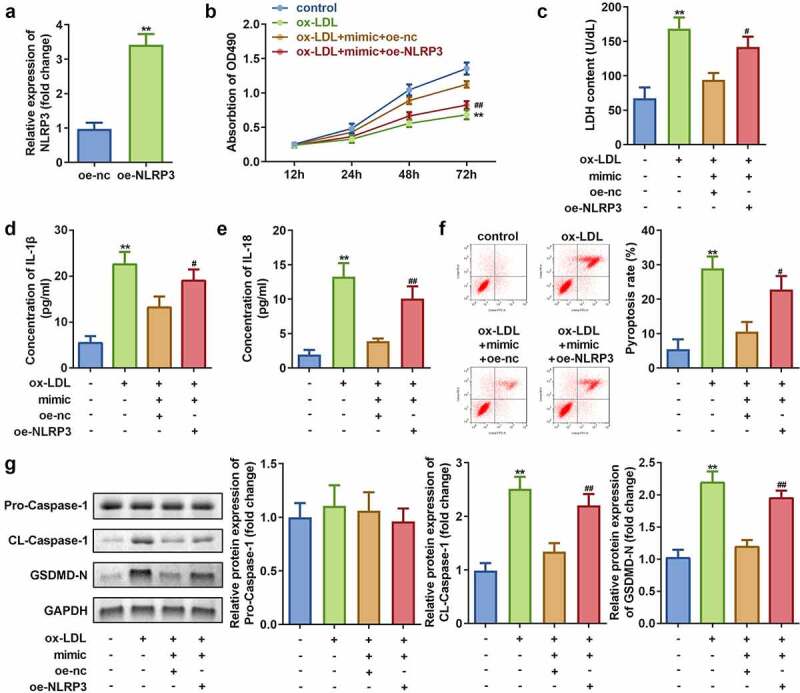
Overexpression of NLRP3 inhibits cell viability while promoting cell injury and pyroptosis in HAECs treated with ox-LDL. (a) Expression of NLRP3; (b) Cell viability; (c) LDH release; (d) IL-1β level; (e) IL-18 level; (f) Cell pyroptosis; and (g) Expression of pro-Caspase 1, cleaved-Caspase 1, GSDMD-N in HAECs cells under indicated conditions. **P < 0.01 versus control, #*P* < 0.05 versus control. oe-nc, blank vector; oe-NLRP3, overexpression of NLRP3; mimic, overexpression of miR-635

To investigate the role of NLRP3 on the effects of ox-LDL exposure to HAECs, NLRP3 overexpression vector was successfully transfected into HAECs as indicated by a significant increase in the NLRP3 expression (Figure 7(a)). Further studies revealed that NLRP3 overexpression decreases the cell viability and improves LDH release in HAECs exposed to ox-LDL as compared to that of the non-treated cells (Figure 7(b)). In addition, overexpression of NLRP3 leads to reversal of reduction in the levels of IL-1β and IL-18 as well as pyroptosis caused by overexpression of miR-635 (Figure 7(d–f)). Moreover, the protein expression of cleaved-Caspase 1 and GSDMD-N were found to be increased upon NLRP3 overexpression, while that of pro-Caspase 1 remained unaffected in HAECs treated with ox-LDL as compared to that in the non-treated cells (Figure 7(g)).

## 4. Discussion

AS-induced cardiovascular diseases are a serious concern to human health [27]. Pyroptosis is a programmed form of cell death associated with the release of inflammatory factors [28]. Here, we showed that dysregulated circ_0090231 contributes to the development of AS. Moreover, circ_0090231 functions as a sponge for miR-635 that further regulates NLRP3 expression. However, knockdown of circ_0090231 leads to reduced cell injury and pyroptosis in AS mimicking conditions. These findings implicate a great potential for the circ_0090231/miR-635/NLRP3 axis for the development of promising treatment strategy for AS.

As circRNAs occupy an important position in post-transcriptional and biological processes, they have been implicated to play vital role in the pathogenesis of various diseases including AS. For example, circ_0044073 activates the JAK1/STAT3 signaling pathway by targeting miR-107 and upregulating the AS-promoting proteins BCL2 and cMYC [29]. In addition, circANRIL knockdown inhibits apoptosis and imparts protection against AS [30]. In the present study, we have demonstrated that circ_0090231 knockdown leads to reduced pyroptosis and cell injury and improved the viability of HAECs treated with ox-LDL, thereby mimicking AS *in vitro* as compared to the control cells (Figure 3). Thus, these findings suggest a promising function of circ_0090231 as a potential therapeutic target for AS.

CircRNAs act as ceRNAs to regulate gene expression and cellular functions by binding to miRNAs. CircRNA_0046367, for example, regulates miR-34a to affect lipid metabolism [31]. To further explore the potential mechanism that regulates AS by circ_0090231, we determined the targets for circ_0090231 and miR-635 through *in silico* predictive analysis. In our study, the expression of miR-635 was a target of circ_0090231. Our results showed that knocking down miR-635 weakens the effect of circ_0090231 inhibition (Figure 5), suggesting that miR-635 is a key factor in the circ_0090231-mediated regulation of AS pathogenesis. Studies have reported that miR-635 acts as an anti-tumor gene in many tumors, for example, miR-635 inhibits tumor proliferation and invasion in non-small cell lung cancer [32], miR inhibit the progression of gastric cancer by targeting KIFC1 [33], and miR-635 is involved in regulation of nasopharyngeal carcinoma as a target gene of circRANBP17 [34]. In this study, miR-635 exerted an anti-inflammatory function and restored the cellular functions of vascular endothelial cells, which is a key factor in maintaining heart function [14].

NLRP3 is an intracellular protein complex that belongs to the nod-like roll receptor (NLR) family and is mainly expressed in innate immune cells, adaptive immune cells, and epithelial cells [35]. Under pathological conditions, NLRP3 can activate Caspase 1, IL-1β, IL-18, and other immune factors [36], thus playing a vital regulatory role in AS [37,38]. In this study, NLRP3 was found to be a target gene of miR-635 and Figure 7 showed that its overexpression led to enhanced cell injury and pyroptosis, as well as reduced cell viability in HAECs treated with ox-LDL as compared to non-treated cells.

## 5. Conclusion

In summary, the findings of this study showed that circ_0090231 is overexpressed in AS. Furthermore, knockdown of circ_0090231 inhibits cell injury and pyroptosis through the miR-635/NLRP3 axis, thereby inhibiting the occurrence and development of AS. These results, thus provide potential targets in the form of circ_0090231/miR-635/NLRP3 axis that can prove useful in the development of novel treatment strategy for AS.

## Supplementary Material

Supplemental Material

## References

[1] Benjamin EJ, Blaha MJ, Chiuve SE, et al. Heart disease and stroke statistics-2017 update: a report from the American heart association. Circulation. 2017;135:e146–e603. 28122885 10.1161/CIR.0000000000000485 PMC5408160

[2] Hansson GK. Inflammation, atherosclerosis, and coronary artery disease. N Engl J Med. 2005;352(16):1685–1695. 15843671 10.1056/NEJMra043430

[3] Ulleryd MA, Prahl U, Borsbo J, et al. The association between autonomic dysfunction, inflammation and atherosclerosis in men under investigation for carotid plaques. PLoS One. 2017;12(4):e0174974. 28376102 10.1371/journal.pone.0174974 PMC5380339

[4] King SM, McNamee RA, Houng AK, et al. Platelet dense-granule secretion plays a critical role in thrombosis and subsequent vascular remodeling in atherosclerotic mice. Circulation. 2009;120(9):785–791. 19687360 10.1161/CIRCULATIONAHA.108.845461 PMC2761818

[5] Ballinger SW, Patterson C, Knight-Lozano CA, et al. Mitochondrial integrity and function in atherogenesis. Circulation. 2002;106(5):544–549. 12147534 10.1161/01.cir.0000023921.93743.89

[6] Ross R, Epstein FH. Atherosclerosis–an inflammatory disease. N Engl J Med. 1999;340(2):115–126. 9887164 10.1056/NEJM199901143400207

[7] Kiechl S, Egger G, Mayr M, et al. Chronic infections and the risk of carotid atherosclerosis: prospective results from a large population study. Circulation. 2001;103(8):1064–1070. 11222467 10.1161/01.cir.103.8.1064

[8] Zychlinsky A, Prevost MC, Sansonetti PJ. Shigella flexneri induces apoptosis in infected macrophages. Nature. 1992;358(6382):167–169. 1614548 10.1038/358167a0

[9] Doitsh G, Galloway NL, Geng X, et al. Cell death by pyroptosis drives CD4 T-cell depletion in HIV-1 infection. Nature. 2014;505(7484):509–514. 24356306 10.1038/nature12940 PMC4047036

[10] Shi J, Zhao Y, Wang K, et al. Cleavage of GSDMD by inflammatory caspases determines pyroptotic cell death. Nature. 2015;526(7575):660–665. 26375003 10.1038/nature15514

[11] Kayagaki N, Stowe IB, Lee BL, et al. Caspase-11 cleaves gasdermin D for non-canonical inflammasome signalling. Nature. 2015;526(7575):666–671. 26375259 10.1038/nature15541

[12] Zhaolin Z, Guohua L, Shiyuan W, et al. Role of pyroptosis in cardiovascular disease. Cell Prolif. 2019;52(2):e12563. 30525268 10.1111/cpr.12563 PMC6496801

[13] Pan J, Han L, Guo J, et al. AIM2 accelerates the atherosclerotic plaque progressions in ApoE-/- mice. Biochem Biophys Res Commun. 2018;498(3):487–494. 29510138 10.1016/j.bbrc.2018.03.005

[14] Zhang Y, Liu X, Bai X, et al. Melatonin prevents endothelial cell pyroptosis via regulation of long noncoding RNA MEG3/miR-223/NLRP3 axis. J Pineal Res. 2018;64:e12449. 10.1111/jpi.12449 29024030

[15] Han Y, Qiu H, Pei X, et al. Low-dose sinapic acid abates the pyroptosis of macrophages by downregulation of lncRNA-MALAT1 in rats with diabetic atherosclerosis. J Cardiovasc Pharmacol. 2018;71(2):104–112. 29095793 10.1097/FJC.0000000000000550

[16] Jeck WR, Sorrentino JA, Wang K, et al. Circular RNAs are abundant, conserved, and associated with ALU repeats. RNA. 2013;19(2):141–157. 23249747 10.1261/rna.035667.112 PMC3543092

[17] Salzman J, Chen RE, Olsen MN, et al. Cell-type specific features of circular RNA expression. PLoS Genet. 2013;9(9):e1003777. 24039610 10.1371/journal.pgen.1003777 PMC3764148

[18] Memczak S, Jens M, Elefsinioti A, et al. Circular RNAs are a large class of animal RNAs with regulatory potency. Nature. 2013;495(7441):333–338. 23446348 10.1038/nature11928

[19] Lin F, Zhao G, Chen Z, et al. circRNAmiRNA association for coronary heart disease. Mol Med Rep. 2019;19:2527–2536. 30720076 10.3892/mmr.2019.9905 PMC6423602

[20] Zhang F, Zhang R, Zhang X, et al. Comprehensive analysis of circRNA expression pattern and circRNA-miRNA-mRNA network in the pathogenesis of atherosclerosis in rabbits. Aging (Albany NY). 2018;10(9):2266–2283. 30187887 10.18632/aging.101541 PMC6188486

[21] Kumar P, Nagarajan A, Uchil PD. Analysis of cell viability by the MTT assay. Cold Spring Harb Protoc. 2018;2018:469-471. 10.1101/pdb.prot095505 29858338

[22] Zhu Z, Li J, Zhang X. Salidroside protects against ox-LDL-induced endothelial injury by enhancing autophagy mediated by SIRT1-FoxO1 pathway. BMC Complement Altern Med. 2019;19(1):111. 31146723 10.1186/s12906-019-2526-4 PMC6543685

[23] Kurien BT, Scofield RH. Western blotting: an introduction. Methods Mol Biol. 2015;1312:17–30. 26043986 10.1007/978-1-4939-2694-7_5 PMC7304528

[24] Unal H. Luciferase reporter assay for unlocking ligand-mediated signaling of GPCRs. Methods Cell Biol. 2019;149:19–30. 30616819 10.1016/bs.mcb.2018.08.001

[25] Torres M, Becquet D, Guillen S, et al. 2018. RNA pull-down procedure to identify RNA targets of a long non-coding RNA. J Vis Exp. 134. DOI: 10.3791/57379. PMC5933463 29708552

[26] Peng H, Sun J, Li Y, et al. Circ-USP9X inhibition reduces ox-LDL-induced endothelial cell injury via the miR-599/CLIC4 axis. J Cardiovasc Pharmacol. 2021;Publish Ahead of Print. DOI:10.1097/FJC.0000000000001104 34269702

[27] Kobiyama K, Ley K. Atherosclerosis. Circ Res. 2018;123(10):1118–1120. 30359201 10.1161/CIRCRESAHA.118.313816 PMC6298754

[28] Zheng Y, Gardner SE, Clarke MC. Cell death, damage-associated molecular patterns, and sterile inflammation in cardiovascular disease. Arterioscler Thromb Vasc Biol. 2011;31(12):666–676. 10.1161/ATVBAHA.111.224907 22096097

[29] Shen L, Hu Y, Lou J, et al. CircRNA0044073 is upregulated in atherosclerosis and increases the proliferation and invasion of cells by targeting miR107. Mol Med Rep. 2019;19:3923–3932. 30864721 10.3892/mmr.2019.10011

[30] Daskalopoulos EP, Dufeys C, Beauloye C, et al. AMPK in cardiovascular diseases. Exp Suppl. 2016;107:179–201. 27812981 10.1007/978-3-319-43589-3_8

[31] Guo XY, Sun F, Chen JN, et al. circRNA_0046366 inhibits hepatocellular steatosis by normalization of PPAR signaling. World J Gastroenterol. 2018;24(3):323–337. 29391755 10.3748/wjg.v24.i3.323 PMC5776394

[32] Zhang Y, Sun Z, Zhang Y, et al. The microRNA-635 suppresses tumorigenesis in non-small cell lung cancer. Biomed Pharmacother. 2016;84:1274–1281. 27810784 10.1016/j.biopha.2016.10.040

[33] Cao FY, Zheng YB, Yang C, et al. miR-635 targets KIFC1 to inhibit the progression of gastric cancer. J Investig Med. 2020;68(8):1357–1363. 10.1136/jim-2020-001438 32753405

[34] Zhou M, Zhang P, Zhao Y, et al. Overexpressed circRANBP17 acts as an oncogene to facilitate nasopharyngeal carcinoma via the miR-635/RUNX2 axis. J Cancer. 2021;12(14):4322–4331. 34093832 10.7150/jca.55794 PMC8176428

[35] Kim JK, Jin HS, Suh HW, et al. Negative regulators and their mechanisms in NLRP3 inflammasome activation and signaling. Immunol Cell Biol. 2017;95(7):584–592. 28356568 10.1038/icb.2017.23

[36] An N, Gao Y, Si Z, et al. Regulatory Mechanisms of the NLRP3 inflammasome, a novel immune-inflammatory marker in cardiovascular diseases. Front Immunol. 2019;10:1592. 31354731 10.3389/fimmu.2019.01592 PMC6635885

[37] Arend WP, Palmer G, Gabay C. IL-1, IL-18, and IL-33 families of cytokines. Immunol Rev. 2008;223(1):20–38. 18613828 10.1111/j.1600-065X.2008.00624.x

[38] Tschopp J. Mitochondria: sovereign of inflammation? Eur J Immunol. 2011;41(5):1196–1202. 21469137 10.1002/eji.201141436

