# Supplementary figures and images for: Circular RNA circ_0090231 promotes atherosclerosis in vitro by enhancing NLR family pyrin domain containing 3-mediated pyroptosis of endothelial cells
Source: Bioengineered. 2021 Nov 30;12(2):10837–48. doi: 10.1080/21655979.2021.1989260 (PMC8809982; doi:10.1080/21655979.2021.1989260)

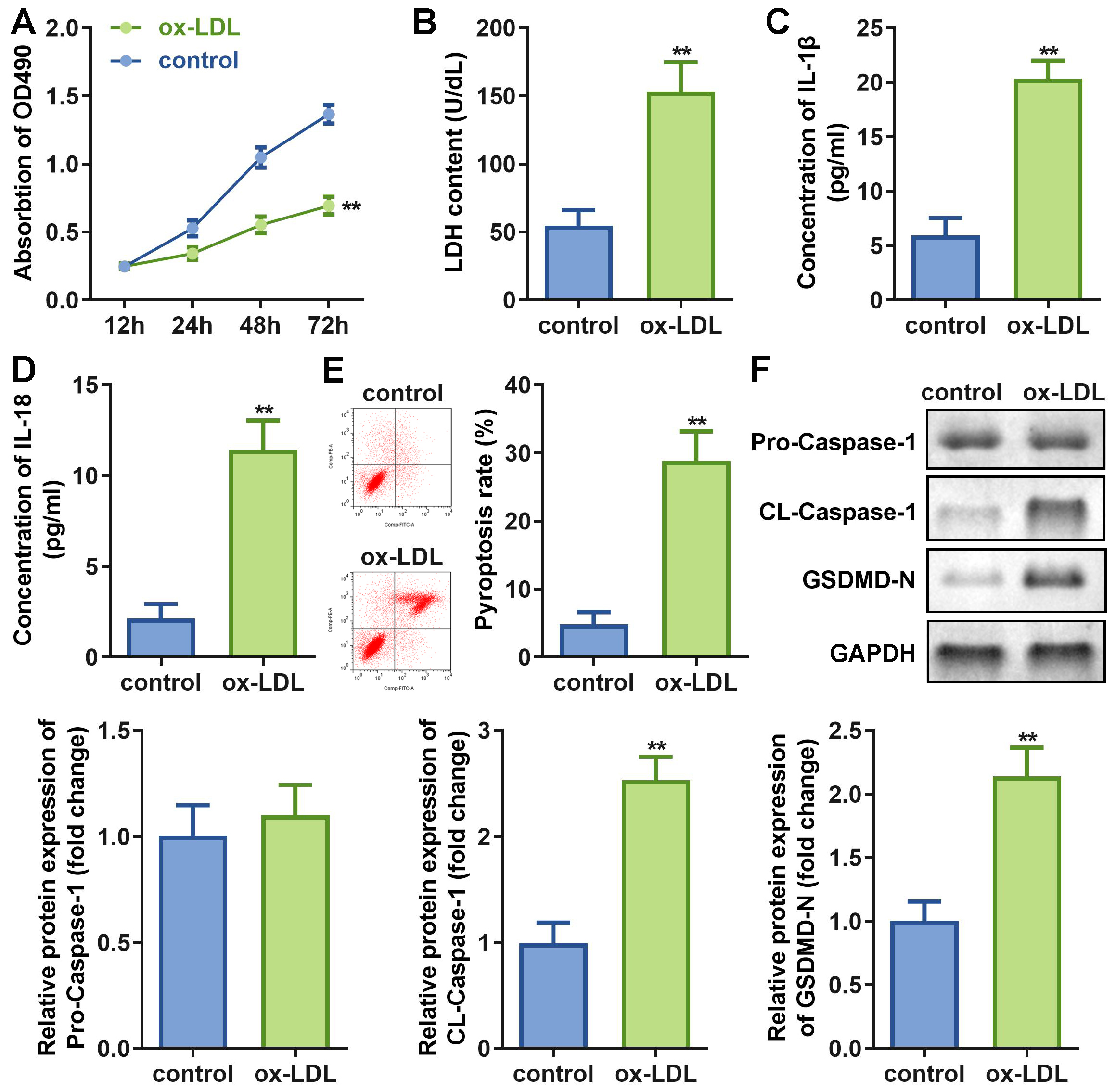

Supplement: Supplemental Material [file KBIE_A_1989260_SM7123.jpg]
